# Effect of different rearing system on eggs production, hatchability, and offspring quality in layer breeders

**DOI:** 10.1016/j.psj.2021.101101

**Published:** 2021-03-11

**Authors:** Krzysztof Damaziak, Marta Musielak, Cezary Musielak, Julia Riedel, Dariusz Gozdowski, Weronika Grzybek

**Affiliations:** ⁎ Department of Animal Breeding, Institute of Animal Sciences, Warsaw University of Life Sciences, Ciszewskiego 8, 02-786 Warsaw, Poland; † Musielak S.A., ISA Brown Reproduction Farm and Hatchery, Niedabyl 49, Poland; ‡ Musielak S.A., ISA Brown Rearing Farm, Topolowa 29, 05-504 Korzeniówka, Poland; § Department of Biometry, Faculty of Agriculture and Biology, Institute of Agriculture, Warsaw University of Life Sciences, Nowoursynowska 159, 02-786 Warsaw, Poland; # Department of Biotechnology and Nutrigenomics, Institute of Genetics and Animal Biotechnology, Polish Academy of Sciences, Jastrzębiec 36 A, Magdalenka, Poland

**Keywords:** layer, breeder, management, reproduction, chicks

## Abstract

This study analyzed the influence of an aviary system, in comparison with battery cages, on rearing and reproduction of parent-stock **(PS)** laying-type chickens. ISA Brown PS chicks were reared for 16 wk in battery cages or in an aviary system. Chickens reared in cages were kept there throughout the rearing period, whereas those reared in the aviary were released after 7 wk. The remaining housing conditions were similar in cages and the aviary. Body weight (**BW**, g), feed intake (FI, g/birds/d), and mortality (%) of birds were monitored during rearing. After the rearing period, the chickens were transferred to 4 litter poultry houses: flock C (in cages) to poultry houses C1 and C2 (total: 2,076 cockerels and 20,450 pullets); flock A (in aviary) to poultry houses A1 and A2 (total: 1,542 cockerels and 16,962 pullets). During the period of reproduction (48 wk), egg production (%), hatching egg production (%), waste egg (%) and litter egg production (%), feed conversion ratio (FCR, g) and water intake (mL) per laid egg, hatching egg, and hatched chick, mortality (week/%), and BW at 17 wk and after reaching 50% laying performance were monitored. Furthermore, during incubation, fertilization rate (%), hatchability (%), and chick quality were recorded. The results showed that aviary rearing was associated with lower FI and higher mortality of chicks up to 16 wk of age. The following effects were also observed for aviary rearing during reproduction: the average egg and hatching egg production were higher, while waste and litter egg production were lower; FCR per laid egg, hatching egg, and the number of hatched chicks were poorer; and water intake for the production of 1 hatching egg and 1 hatched chick was lower. In the case of flocks A, higher mortality and BW at 17 wk of age were recorded for both sexes. They were characterized by higher relative egg fertilization, but lower hatchability due to the higher share of unhatched eggs. No influence of PS flock rearing system on chick quality was observed. The obtained results indicate that the aviary rearing system can be recommended for PS laying-type flocks. However, future research should consider the impact of a different diet having higher energy concentration on PS flocks reared in aviaries and develop methods for counteracting higher mortality in these systems. This is particularly significant for roosters because too few roosters in flock may contribute to lower egg fertilization and higher embryonic mortality.

## INTRODUCTION

The European Union Council Directive 1999/74/EC (European Communities, 1999), which has been effective since January 2012, prohibits husbandry for laying hens in traditional battery cages. According to above-mentioned EU Council Directive, battery hens can only be done in so-called “enriched cages” However, cages are still most applied for rearing laying hens, in the case of both commercial and parent breeding flocks **(PS)**, as they allow simultaneous rearing of many chickens, with lower handling costs, and maintaining a strict sanitary regime. On the other hand, cages offer only minimal opportunities for the hens to express their natural behaviors and affect their welfare. Due to these limitations, keeping of hens in cages is objected by animal rights activists and consumer groups, and hence, changes in the rearing sector can be expected in the future. An alternative to cage keeping is noncage systems, typically multitier aviaries (Campbell et al., 2016). Evidence shows that an aviary system, in which hens can move on a larger area, enhances their welfare by motivating them to exhibit their natural behaviors, such as perching, nesting, dust bathing, and foraging (Ali et al., 2016).

According to van de Weerd and Elson (2006), chickens should be raised in an environment similar to where they will be maintained as adults. This helps to reduce the adaptation problems and is particularly significant in the case of PS flocks, which are typically moved to floor systems. Therefore, aviary rearing may be a favorable alternative to cage rearing. It has been observed that exposing chickens to a complex environment during rearing influences the activation of the hypothalamic–pituitary–adrenal axis and reduces anxiety in adult birds (Johnsen et al., 1998; Brantsaeter et al., 2016). As a result, chickens react differently to stress, depending on the experiences obtained during the rearing period (Jones et al., 1996; Moe et al., 2010). One of the reasons provided for rearing pullets in aviaries is to replicate their natural habitat in this period of life. Aviaries allow the birds to move to different tiers, which enables the development of 3-dimensional spatial awareness, necessary for a smooth transition to the laying period (Alexander, 2019). Furthermore, an aviary system reduces the frequency of laying floor eggs and the occurrence of cloacal cannibalism (Gunnarsson et al., 1999; Damaziak et al., 2020). However, it is associated with a higher incidence of mechanical injuries, including keel bone fractures, which are more commonly observed in cageless systems, especially multilevel facilities, compared to cage systems (Riber et al., 2018).

Unlike commercial laying hen flocks, no information is available for PS flocks regarding the impact of aviary systems on rearing results or the later reproduction. Generally, the level of welfare of PS flocks is known to be lower than commercial flocks, and PS flocks are less capable of dealing with anxiety and stress (Haas et al., 2013). This can be attributed to numerous complex factors, among which the most mentioned are genetic predisposition, maintaining of both sexes together, and following a strict sanitary regime, and therefore, strongly reducing the contact of flocks with humans (Freire and Cowling, 2013). According to Haas et al. (2013), it is necessary to consider the human–bird relations and flock size during rearing. Limited contact with humans increases the anxiety of birds (Haas et al., 2013), and keeping poultry in small flocks can negatively impact the results of PS production. The above conditions are observed typically during cage rearing of chickens. In aviary system, cockerels and pullets are kept in large flocks, and also more frequently come in contact with humans. This is particularly significant for the rearing period, during which the birds are released from cages and taught to return at night by means of light management or manual catching. Although considered as labor-intensive, this treatment creates a stress-free environment for chicken and develops human–bird relationship.

The objective of this study was to analyze the influence of the rearing environment on growth, water and food intake, reproduction, and chick quality of choosen strain of layer breeders. Considering that the earlier study by Damaziak et al. (2020) suggested that releasing PS chicks during rearing outside of aviary sections is enough to favor better adaptation of adult hens to the conditions of barn production system, a hypothesis was formulated that aviary vs battery cages rearing of PS hens might facilitate flock management in the laying period and result in a larger number of high-quality chicks.

## MATERIALS AND METHODS

### Ethical Approval

Approval of the Animal Care Committee was not necessary for this study, as all the required information was obtained from the existing databases.

### Juvenile Parental Flock Rearing and Housing Conditions

**Figure 1:**
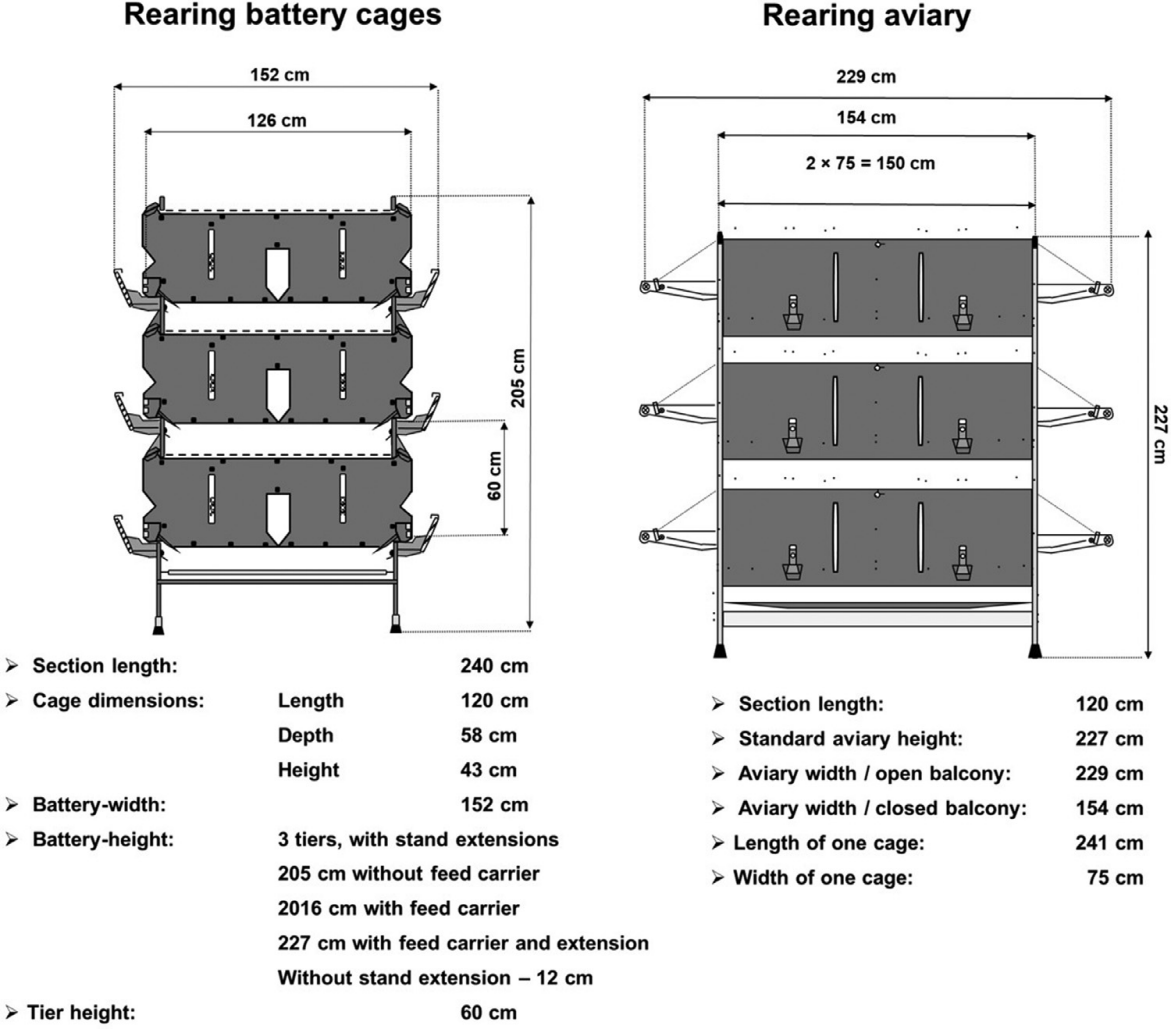
Scheme of construction of rearing battery cages and aviary system.

One-day-old PS ISA Brown chicks (including 3,925 cockerels and 38,550 pullets) were purchased from ISA, Hendrix-Genetics (Netherlands) and transported to Musielak S.A., ISA Brown Rearing Farm (Korzeniówka, PL). Some of them (2,244 cockerels and 20,700 pullets) were introduced into 3-level battery cages (Orginal Specht Poultry Equipment GmbH & Co. KG, Dassendaler Weg, Sonsbeck, Germany), while others (1,681 cockerels and 17,850 pullets) were kept in a 3-level aviary system (Hellmann Poultry GmbH & Co. KG, Kopernikusstr, Vechta, Germany). The dimensions of the battery cages and the aviary system are presented in Figure 1.

*Cage rearing* When stocking the rearing facility, the chicks (1-d old) were placed in the middle and top levels of the battery cages (males and females separately). About 50 cockerels or 65 pullets were introduced into each cage. At 8 d of age, the stock was reduced to 30 chickens in a single cage, and some of the birds were moved to the lower level, still separated by sex. From 10 d of age till the end of rearing, the stocking density was maintained at about 230 cm^2^/bird. Feed was distributed by an automatic feeder line outside the cages. Four nipple drinkers were installed in all levels of the cages. Manure belt was located under each level.

*Aviary rearing* Chick rearing in aviary system taking into account the stocking and zootechnical treatments was conducted in compliance with the methodology described earlier by Damaziak et al. (2020). The 1-d old chicks were first placed in the middle level in closed sections. Approximately 105 cockerels or 113 pullets were introduced into a single section. At 10 d of age, half of the chicks were moved to the lower level. Until 7 wk, all the chickens were reared under similar conditions (pullets and cockerels separately) in closed sections, where they could not move between the levels and had no access to the floor area. During this period, stocking density was maintained at about 170 cm^2^/bird for cockerels and 160 cm^2^/bird for pullets. From the 7th wk onward, all the chickens were released from the aviary sections and were allowed to use the floor area and all levels (pullets and cockerels together). After the third-level chickens were released, the usable area increased to 245 cm^2^/bird. The birds were trained to return to the aviary through systematic switching on and off of LEDs in the sections, which enabled the equal distribution of chickens in all 3 levels. To facilitate the chickens to move to different levels, every third balcony was opened to form stairs. The ability of birds to return to their sections was monitored for the first 10 d, and chickens that stayed on the floor were manually introduced into the sections. Feed was distributed to the birds by a chain feed line that ran centrally through each section. Four nipple drinkers were installed at each level of sections.

All chickens, independently from rearing system were fed *ad libitum* with the same diets in 2-phase system. From the 1st day to the 7th wk, diet included 18.5% crude protein, 2,880 ME kcal/kg and from of the 8th to 16th wk of age - 15.5% crude protein, 2,790 ME kcal/kg. Feed intake (FI) was calculated as the amount of day feed consumed (g) by each chicken. In addition, the number of dead and defective birds was recorded on a daily basis, from which percentage mortality in individual weeks and progressive mortality were calculated. During 7, 9, 12, and 13 wk of rearing, the body weight (BW) of 160 chickens randomly selected from different cages and aviary levels was controlled. The rearing conditions, including the light program and the temperature during the 16 wks of rearing, were in line with the recommendations of Hendrix-Genetics (2014). Day length was gradually shortened from 22 h in first day to 14 in 16 wk of age and temperature was reduced from 33.5°C on the 1st day to 20.5°C in the 7th wk.

### Laying Period and Reproduction Results

After 16 wk, all reared chickens were transported to ISA Brown Reproduction Farm and Hatchery (Niedabyl 49, Poland) and introduced into 4 identical poultry houses. Chickens raised in cages were introduced into poultry houses C1 and C2, and those reared in the aviary system into A1 and A2. From the cage-reared flock, 2,076 roosters and 20,450 hens were subjected to reproduction (1,050 roosters and 10,080 hens to poultry house C1 and 1,026 roosters and 10,370 hens to C2), while from the aviary-reared flock, 1,542 roosters and 16,962 hens were used for reproduction (780 roosters and 8,530 hens to poultry house A1 and 762 roosters and 8,432 hens to A2).

The rearing conditions of the studied flocks during reproduction, including: building equipment, stocking, lighting and feeding program were similar to those described earlier by Damaziak et al. (2020). Buildings in which the flocks were maintained had floor and grate structure with finely chopped straw as litter, equipped with central automatic nests, automatic drinker and feeder lines (Big Dutchman, Germany). Lighting duration was set at 15.0 to 16.5 h/day with average intensity of 25.6 lx. Hens and roosters were fed *ad libitum* with the same diet containing 17.0% crude protein, 2,820 ME kcal/kg, 4.69% crude fiber, 3.45% crude fat, and 11.6% crude ash.

In the subsequent week after combining the flocks (17 wk of age), the BW of hens and cockerels was controlled. The next BW control was carried out when 50% laying performance was achieved (at 22 wk of age). For this purpose, 15 roosters and 100 hens were randomly selected from different areas of the poultry houses. Throughout the reproduction period (48 wk), the eggs production was controlled, including total laid eggs, eggs laid on litter, hatching eggs, and waste eggs. A hatching egg was defined as the egg intended for hatching and which met the following criteria: weight 52 to 75 g, presence of clean shell, and absence of any damage or deformation. Waste eggs were defined as those not meeting the above criteria. Daily feed (kg) and water (L) uptake were controlled, and mortalities and rejections were documented. Based on these parameters, the mean egg production and mortality were calculated for the entire laying period for both flocks reared in the same system Laying performance and cumulative mortality curves were plotted for individual flocks (C1, C2, A1, A2). In addition, feed conversion ratio (FCR, g) and water intake (mL) per laid egg, hatching egg, and hatched female were calculated collectively for the flocks reared in the same system.

### Hatching and Chick Quality

Chick hatching control was conducted in a commercial hatchery and in accordance with the standard assumed for laying hens. The hatchery was equipped with setters (Petersime 576, Zulte, Belgium) and hatchers (Petersime AirStreamer 12S, Zulte, Belgium). All procedures regarding the handling of the hatching eggs and egg incubation conditions were standard, which was described in detail by Damaziak et al. (2020). For the purposes of this study analogous hatching in 27, 32, 39 wk of laying were performed for each flock. The total number of eggs analyzed in the study was 19,200 for flock C and 23,850 for flock A. The number of eggs subject to study depended on the sizes of commercial hatching. In order to avoid the effect of egg location in the incubator, only eggs from the fifth from the top and the fifth from the bottom hatching tray were selected for analysis from each trolley (Supplementary Figure S1). The analysis of the content of selected hatching trays after 21 days of incubation was used to calculate hatching indicators, including: hatching of all chicks from the laid eggs, apparent fertilized eggs, and hatching of female chicks from the laid eggs.

Assessment of the quality of hatched female chicks was conducted during each hatching. The assessment was carried out with the use of method developed by Tona et al. (2003), modified by Damaziak et al. (2020). The maximum score for the highest quality chick was set at 100 points. Scale between 0 to 20 points was used to assess activity, navel area, remaining yolk and legs; from 0 to 10 points for eyes appearance; from 0 to 5 points were used to assess fluff appearance and remaining membrane. The ratio of chicks with a score of 100 (%), average score of all chicks, and average score of chicks with a score <100 were calculated for both flocks. Sixty five female chicks were selected for assessment at random from each flock for each hatching date. As a consequence, the total number of chicks assessed was 780.

### Statistical Analysis

The results were presented using descriptive statistics such as means or percentages. The means were compared between the aviary system and cages using analysis of variance **(ANOVA)**. Within group variability was presented as RMSE (Root mean square error) for residuals based on ANOVA model. Multivariate analysis of variance **(MANOVA)** was carried out for multivariate comparisons of selected groups of variables. Both ANOVA and MANOVA were performed according to the following linear model:

$$Y_{ijk}=\;\mu +\alpha_{i}+\;\beta_{j}+\;Y_{k}+\;\varepsilon_{ijkt}$$

where *Y* is the dependent variable; α is the effect of the rearing system; β is the effect of the flock (A, C: 1, 2); γ is the effect of the subsequent day; and ε is the error term. The β and γ effects were not included in the analysis for all traits because not all the variables were evaluated for 2 flocks with 1 treatment (A, C: 1, 2) and subsequent days.

The proportions—final results presented in percentages without replications—were compared between the aviary and cages using Pearson's chi-squared test. The rate of mean egg production in subsequent days was modeled using Yang's model (Yang et al., 1989) as follows:

$$Y_{t}=\;\frac{ae^{-xt}}{1+e^{-c\left(t-d\right)}}$$

where *Y_t_* is the estimated rate of egg production; t is the subsequent day of laying; *a, b, c,* and *d* are the model parameters; and e is the mathematical constant which is approximately equal to 2.718. The parameters of Yang's model are presented in detail in Supplementary Table S1.

The mean litter egg rate for subsequent days was estimated using polynomial cubic functions as follows:

$$y=a+bt+ct^{2}$$

where *Y* is the estimated litter egg rate; *a, b,* and *c* are the model parameters; and t is the subsequent day of laying.

The means for hatching egg rate and waste egg rate for subsequent days were estimated using the 10-d moving average. Statistical analyses were performed using Statistica 13 and Excel 2016 software (TIBCO, 2017). The significance level was set at 0.05 for all the analyses.

## RESULTS

### Rearing Period

**Figure 2:**
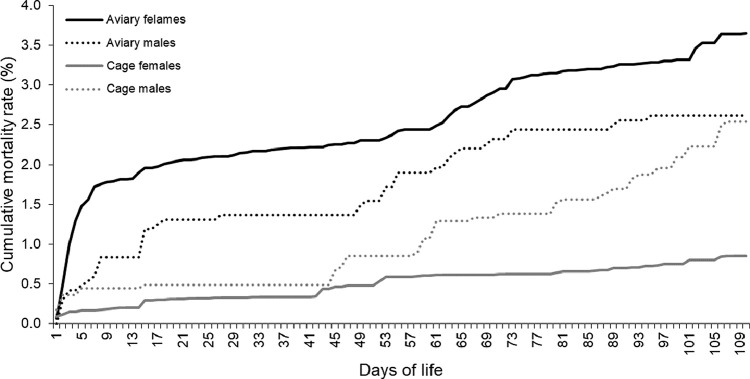
Effect of PS rearing in battery cages and aviary system on the cumulative mortality of cockerel and pullet in the rearing period.

The chicken BW controlled on 7, 9, 12, and 13 wk of age did not differ between the flocks A and C (Table 1). It was observed that cage rearing resulted in higher FI by 3.0 g/birds/d (*P* < 0.05) and lower pullet mortality by 2.8% compared to aviary rearing (*P* < 0.001; Table 1 and Figure 2). On the other hand, the mortality of cockerels was not influenced by the rearing system (*P* = 0.420; Table 1 and Figure 2).

**Table 1:** Effects of aviary and cage rearing on chicken BW, daily FI, and mortality.

Parameter		Aviary		Cages		RMSE	*P*-value* (aviary vs cages)
N^b^		22,644		19,431			
BW (g)	7 wk	686.1^a^		691.1^a^		99.7	0.656
	9 wk	890.1^a^		895.8^a^		84.8	0.551
	12 wk	951.4^a^		968.4^a^		102.7	0.139
	13 wk	1,050.0^a^		1,057.2^a^		83.0	0.439
FI, g/birds/d		46.1^a^		49.1^a^		21.1	
Mortality, %		Cockerels	Pullets	Cockerels	Pullets		<0.001 (female), 0.420 (male)
		2.78^a^	3.65^a^	2.54^a^	0.86^a^		

Abbreviations: ANOVA, analysis of variance; BW, body weight; FI, feed intake; RMSE, root mean square error based on ANOVA for residuals.

a Means within a column with same letters are similar (*P* < 0.05).

b Initial number of cockerel and pullet chickens—aviary: 2,244 cockerels and 20,700 pullets, cages: 1,681 cockerels and 17,850 pullets.

⁎ *P*-value based on ANOVA for means and based on chi-squared test for percentages (for mortality).

### Reproduction Period—Egg Production, FCR, BW, and Mortality

**Figure 3:**
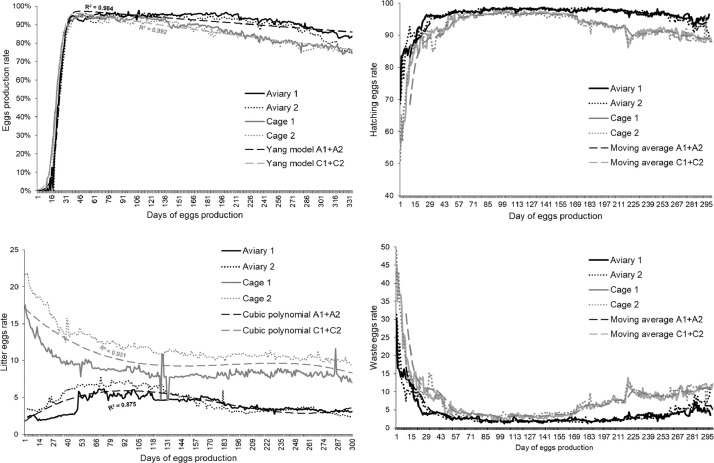
Effect of PS rearing in battery cages and aviary system on average, hatching, litter, and waste egg production.

Compared with cage-reared hens, those reared in aviary showed higher average egg production by 4.3% during 48 wk (*P* < 0.001; Table 2). The course of laying performance (Figure 3) can be described using the following equations based on the average egg production for subsequent days estimated with Yang's model:

Eggproductionafteraviaryrearing=[0.995*exp(d*(0.00043))]/[1+exp(0.285*(d23.99))]

Eggproductionaftercagerearing=[0.997*exp(d*(0.00079))]/[1+exp(0.267*(d21.25))]

**Table 2:** Production results of PS after rearing in aviary and cages.

				Aviary^b^	Cages^c^		
Parameter				Mean or %	Mean or %	RMSE*	*P*-value*
N^d^			Roosters	1,542	276		
			Hens	16,962	20,450		
Average egg production (%)				85.6^a^	81.3^a^	4.1	<0.001
Hatching egg production (%)^e^				96.3^a^	92.3^a^	1.8	<0.001
Waste eggs (%)^f^				3.7^a^	7.7^a^	1.8	<0.001
Litter egg production (%)^g^				4.3^a^	10.4^a^	1.8	<0.001
Together: Average egg production (%); hatching egg production (%); waste eggs (%):							
MANOVA-based *F*-ratio = 2,064.3							<0.001
FCR (g) for:							
	One laid egg			152.7^a^	133.7^a^	14.3	<0.001
	One hatching egg			158.8^a^	145.5^a^	15.8	<0.001
	One hatched female			407.2^a^	397.2^a^	45.2	<0.001
Together FCR (g) for: one laid egg; one hatching egg; one hatched female:							
MANOVA-based *F*-ratio=611.1							<0.001
Water used (mL) for:							
	One laid egg			263.0^a^	263.1^a^	18.7	0.101
	One hatching egg			273.3^a^	286.5^a^	21.7	<0.001
	One hatched female			700.8^a^	782.7^a^	70.5	<0.001
Together water used (mL) for: one laid egg; one hatching egg; one hatching female:							
MANOVA-based *F*-ratio=365.3							<0.001
Mortality^h^		Roosters	(wk)	0.19^a^	0.05^a^	0.16	<0.001
			(%)	10.0^a^	2.1^a^		<0.001
		Hens	(wk)	0.18^a^	0.20^a^	0.17	0.384
			(%)	11.2^a^	8.5^a^		<0.001
BW in 17 wk (g)		Roosters		1,808^a^	1,705^a^	156.0	0.006
		Hens		1,249^a^	1,215^a^	107.3	0.002
BW in 22 wk (g)		Roosters		2,300^a^	2,253^a^	119.3	0.170
		Hens		1,691^a^	1,676^a^	102.5	0.149

Abbreviations: ANOVA, analysis of variance; BW, body weight; FCR, feed conversion ratio; MANOVA, multivariate analysis of variance; PS, parent stock; RMSE, root mean square error based on ANOVA for residuals.

a Main effect—significantly different at *P* < 0.05.

b Aviary = PS flock rearing in aviary.

c cages = PS flock rearing in cages.

d number of reproduction roosters and hens.

e eggs intended for hatching (quality standards of hatching eggs).

f eggs not intended for hatching (without hatching standard).

g litter eggs intended for hatching (quality standards of hatching eggs).

h weekly average mortality and total mortality (%) of production.

⁎ *P*-value based on ANOVA for means and based on chi-squared test for percentages (for mortality).

These results demonstrated that A hens (both A1 and A2 flocks) had better resistance after the peak of laying, until the end of production. Similarly, flocks A exhibited higher hatching egg production throughout the laying period in comparison with flocks C by 4.0% (*P* < 0.001; Table 2). In particular, the production of hatching eggs was observed to be higher (flocks A) before the laying peak and from 25 wk till the end of production (Figure 3). This was attributed to the considerably lower production of waste eggs in this period (Figure 3). In addition, throughout the production period, the mean waste egg production was lower in flocks A (by 4%) compared with flocks C (*P* < 0.001; Table 2). In the entire production period, flocks A1 and A2 laid a lower number eggs of litter (Figure 3). The mean production of litter eggs throughout the laying period was lower in A hens than C hens by 6.1% (*P* < 0.001; Table 2). Analysis of all 4 traits together (average, hatching, waste, and litter egg production) confirmed a considerable advantage in the case of A hens in comparison with C hens (*P* < 0.001; Table 2).

Compared with flocks A, flocks C (roosters and hens together) required a lower amount of feed for producing 1 laid egg (by 19.0 g), 1 hatching egg (by 13.3 g), and 1 hatched female (by 10.0 g) (all *P* < 0.001; Table 2), but more water for producing 1 hatching egg (by 13.2 mL) and 1 hatched female (by 81.9 mL) (both *P* < 0.001; Table 2). However, the flocks showed no difference in water intake for the production of 1 laid egg (*P* = 0.101; Table 2). Multivariate analysis of FCR and water intake further confirmed the differences between the flocks (both *P* < 0.001; Table 2).

**Figure 4:**
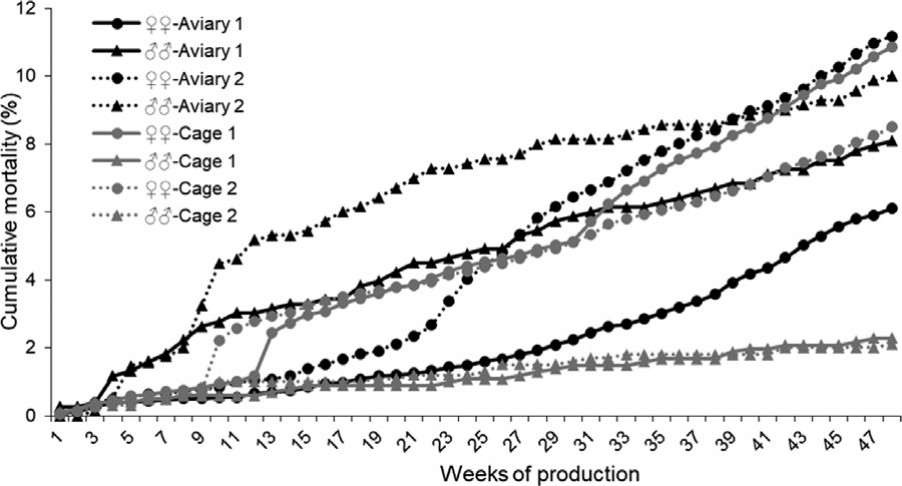
Effect of PS rearing in battery cages and aviary system on the cumulative mortality of roosters and hens in the reproduction period.

Average weekly and cumulative (%) mortality of roosters were higher in flocks A compared to flocks C (*P* < 0.001; Table 2 and Figure 4). However, no difference was found in the weekly mortality between flocks A and C (*P* = 0.384), while similar to roosters, the cumulative mortality was higher in flocks A (*P* < 0.001; Table 2 and Figure 4).

BW monitoring during the 17th week of age, that is, the 1st week after the transfer of flocks to production poultry houses, showed an increase in weight in both roosters (*P* = 0.006) and hens (*P* = 0.002) from flocks A as compared with flocks C (Table 2). After 5 wk of production, the repeated control did not reveal any considerable difference in BW for both sexes between the flocks (*P* > 0.05; Table 2).

PS flock rearing system did not seem to influence the egg hatchability (*P* = 0.102; Table 3). However, flocks A exhibited a higher fertilization rate by 3.3% (*P* < 0.001) as well as a higher embryo mortality or higher number of unhatched eggs by 2.2% (*P* < 0.001) in comparison with flocks C. As a consequence, hatchability from fertilized eggs in flocks C was higher than flocks A by 2.0% (*P* < 0.001; Table 3). Furthermore, compared with flocks C, there were 1.5% more chicks in flocks A (*P* = 0.003; Table 3).

**Table 3:** Hatching results of PS after rearing in aviary and cages.

	Aviary^b^	Cages^c^		
Parameter	Mean	Mean	RMSE	*P*-value*
Set eggs (N)	23,850	19,200		
Hatchability of set eggs (%)	79.1^a^	78.1^a^	5.1	0.102
Apparent fertility (%)	91.1^a^	87.8^a^	3.6	<0.001
No. of hatched chicks (%)	11.9^a^	9.7^a^	3.8	<0.001
Hatchability of apparent fertile eggs (%)	86.9^a^	88.9^a^	4.3	<0.001
Hatched females (%)	39.1^a^	37.6^a^	4.2	0.003

Abbreviations: ANOVA, analysis of variance; PS, parent stock; RMSE, root mean square error based on ANOVA for residuals.

a Main effect—significantly different at *P* < 0.05.

b Aviary = PS flock rearing in aviary.

c cages = PS flock rearing in cages.

⁎ *P*-value based on ANOVA.

No impact of the rearing system could be found on the quality of chicks (Table 4).

**Table 4:** Chick quality of PS after rearing in aviary and cages.

	Aviary^a^	Cages^b^		
Parameter	Mean or %	Mean or %	RMSE	*P*-value*
Chicks with score = 100 (%)	83.8	81.0		0.308
Average score of all chicks	98.79	98.88	4.26	0.781
Average score of chicks with score <100	92.56	94.09	8.21	0.276

Abbreviations: ANOVA, analysis of variance; PS, parent stock; RMSE, root mean square error based on ANOVA for residuals.

Main effect—significantly different at *P* ≤ 0.05.

a Aviary = PS flock rearing in aviary.

b cages = PS flock rearing in cages.

⁎ *P*-value based on ANOVA for means and based on chi-squared test for percentages (for chicks with score = 100).

## DISCUSSION

Although numerous studies have analyzed the impact of different systems used for laying hens keeping on their production results, research on the influence of the rearing system is rare (Moe et al., 2010; Tahamtani et al., 2014). There are no data on FI and mortality of juvenile chickens. Even in the best-managed flock, a certain number of chickens do not reach the laying period and die, which can be due to early post hatching mortality, mechanical injuries, and aggression within the flock (Merle et al., 2009). The results obtained in the present study suggest that the aviary system of rearing can possibly be associated with increased mortality. It must be emphasized that in this study, mortality was found to be practically higher in the aviary system throughout the rearing period, and thus, it is not only linked to the release of chickens outside sections after 7 wk of age. The structure of the aviary itself may probably have a considerable impact, followed by the course of the inside feed line, which is located outside in conventional battery cages. Such an arrangement, which is unavoidable in an aviary system, results in more frequent mechanical injuries and hence higher mortality. At the same time, the internal course of feed line can limit feed scattering outside the birds’ range, and thus reduce FI. In this study, FI is expressed in g/birds/d, and its conversion into cumulative feed (kg) (FC) showed that flocks C were characterized by correct FC (5.50 kg), while flocks A had low FC (5.16 kg), as Hendrix-Genetics (2020) has recommended that FC should be 5.50 kg at 16 wk of life. However, it should be noted that the field observations, which formed the basis for the above results, do not allow a concomitant analysis of numerous replications, and the high number of hens in examined flocks points to the tendencies that would require verification in future research.

A significant observation of the present study is that aviary rearing of PS flocks contributed to better egg production in comparison with cage rearing. An earlier study on commercial hens by Tahamtani et al. (2014) revealed that aviary rearing resulted in earlier sexual maturity and higher egg production in the first 2 months, but rapidly decreased the laying performance of hens compared to cage-reared birds. This could be linked to the fact that greater living surface during rearing increases the concentration of sexual steroids during laying (Janczak et al., 2009). However, in our study, the course of laying did not confirm these results. No differences were observed between flocks A and C in the date of start of laying and beginning laying rate. The factor associated with the better general production of eggs in flocks A was the lower decrease of laying performance after the peak. It has also been shown that compared to commercial flocks, PS flocks reacted differently and favored aviary rearing. Furthermore, in the study of Tahamtani et al. (2014), commercial chickens that were reared in cages and aviary systems were moved to battery cages, whereas in the present study, the adult birds were reared in a litter system. Flocks A were moved to an environment that closely resembled the rearing conditions than where flocks C were reared, which is contrary to the study of Tahamtani et al. (2014).

Naturally, the level of egg production itself is one of the most important indicators of the economic efficiency of hens; however, the applicability of eggs for hatching is more significant in the case of reproduction flocks. The basic criterion that determines the hatching of an egg is its weight and shell thickness, and, the absence of shell damage and deformities. In this study, flocks A1 and A2 produced more eggs in total and a lower number of waste eggs, and therefore, more eggs were available for incubation, compared to flocks C1 and C2. This was related to the next analyzed indicator—the number of eggs laid on litter. The sole fact that an egg was laid outside the nest on the litter does not disqualify it as a hatching egg. However, eggs laid on litter are far more frequently subject to mechanical damage or are so severely soiled that they are eliminated during selection for incubation (van den Brand et al., 2016). Berrang et al. (1997) showed that even 1 in 10 eggs laid outside the nest was not fit for hatching. The number of eggs laid outside the nest not only decreased the quality of eggs but also increased the labor intensity during collection. This greatly affected the producers of commercial laying flocks, and therefore, they moved the hens to aviary systems after rearing in battery cages. For instance, Matthews and Summer (2015) estimated 3 cents/dozen eggs as the cost incurred for picking eggs up from the floor due to keeping adult flocks in aviaries. Thus, our study suggests that laying eggs on litter can be limited by using an aviary rearing system for PS flocks. Furthermore, the earlier study by Damaziak et al. (2020) demonstrated that releasing chicks at 7 wk of rearing outside of the closed aviary sections is enough to reduce the number of litter eggs in the reproduction period. Although numerous factors can cause egg laying outside the nest, such as time of hen transfer, nest location, light intensity, and even genetic factors, the rearing system remains the most important one (Sørensen et al., 2017). Hence, due to a greater surface area and the possibility to move to different levels, rearing in aviary conditions facilitates the development of spatial orientation and awareness in chickens (Alexander, 2019). This experience enables hens to seek nests, while jumping on the edge of a nest consequently provokes them to use it.

The considerably lower number of eggs laid on litter in flocks A in comparison with flocks C suggests the differences in the behavior of chickens reared in different systems. Perhaps, these behaviors could be responsible for the higher feed requirement of flocks A for egg production, including hatching egg and 1 hatched female, than flocks C. Luiting et al. (1991) showed that the variability in energy demand is the main factor causing differences in FI between hens with a similar level of egg production. Similarly, Clark et al. (2019) analyzed the individual behaviors of hens and concluded that birds with lower feed efficiency (i.e. higher FCR) spent more time on feeding, covered greater distances in the poultry house, and rested for shorter periods. As mentioned earlier, an aviary system predisposes chickens to exhibit such behaviors, and hens that have learned certain models during the rearing period reproduce them at an older age (Ali et al., 2016). Thus, in the present study, greater energy expenditure seemed to be associated with greater physical activity which resulted in higher FCR in A hens, despite higher egg production. In addition, greater mortality in flocks A could have contributed to deteriorated FCR. Although the numbers of chickens in the studied flocks were monitored and regularly updated, some portion of the feed was consumed by those birds that died on the given day. Weeks et al. (2016) showed that FCR calculated per egg weight (kg/kg) increased from 2.9 with theoretical zero hen mortality to 4.1 with mortality amounting to 25%. However, there is no information in the literature regarding lower water requirement by A hens for the production of 1 hatching egg and 1 hatched female. Water consumption largely depends on the temperature and air humidity in the poultry house and is also determined by the form and quality of feed (Xin et al., 2002). In the above study, these factors were uniform for all flocks and therefore could not significantly affect water consumption. This may be related to the production size and egg weight. For laying heavier eggs, hens require an increased amount of water. However, egg weight was not monitored in the discussed study, and hence, this assumption should be confirmed or challenged in future research.

The basic target of reproduction farms is to achieve the highest possible number of eggs that could be qualified as hatching eggs. In this study, the higher fertilization rate observed in flocks A than flocks C is therefore a significant result, yet the higher embryo mortality observed at the end of the incubation period raises doubts about the efficiency of aviary rearing in the case of PS flocks. Better fertilization rate might have resulted from keeping hens and roosters from flocks A together from the 7th week of age, while in flocks C the sexes were combined only after rearing. Leonard et al. (1993) examined the impact of PS White Leghorn rearing conditions in their study and demonstrated that roosters that were reared separately from hens seemed to exhibit impaired sexual behaviors and elevated aggression compared to those reared in flocks with hens from the beginning. However, a reverse situation could be expected due to considerably higher mortality of roosters in flocks A because the sex ratio in the flocks was altered. Nevertheless, hens can lay fertilized eggs after 1 mating for up to 14 d (Bakst et al., 1994). Therefore, presumably, the decreased number of roosters in flocks A was not severe enough to exceed the time between copulation. Less frequent mating may however result in the mobilization of “old” sperm cells, which are stored in sperm-host glands for fertilization (Hocking and Bernard, 2000), and fertilization of an egg cell by a sperm cell stored for a longer period increases embryo mortality in a linear manner (Lodge et al., 1971).

The objective of maintaining laying hens at the PS flock level is to obtain female chicks for commercial production. The present study compared the reproduction efficiency of flocks A and C and calculated the number of hatched females. A considerably higher percentage of female chicks observed in flocks A seems to be promising, but the result should not be overestimated. Although Damaziak et al. (2020) observed increase of hatched females by 0.7% than PS flock reared in open aviaries, the sex of chicks is genetically determined by the inherited sex chromosomes. Hence, the environmental conditions of the sires and dams have little or no effect on the sex (Chue and Smith, 2011). Thus, the 1.5% higher number of hatched female chicks observed in flocks A may be accidental or might have stemmed from thus far unknown mechanisms and requires explanation.

The last stage of the study involved the assessment of chick quality. The quality of hatched chicks is a factor determining the rearing efficiency and later production of adult flocks. Therefore, the absence of differences between flocks A and C can be considered as a significant finding and indicate that aviary rearing of PS flocks does not result in the reduction of chick quality.

## CONCLUSION

The results of this study confirm the original hypothesis that aviary rearing of PS laying-type flocks facilitates flock management in the laying period as it lowers the percentage of eggs laid on litter. However, no increase was observed in the number of hatched chicks, which could be related to the higher mortality of parental roosters. Because too few roosters in flock may contribute to lower egg fertilization and higher early embryonic mortality. In general, the aviary rearing system results in higher mortality of both juvenile and adult chickens. Other limitations associated with this system are the poorer FCR during the laying period and higher embryo mortality. However, it appears that these could be overcome in the future by modifying the energy value of feed and developing methods for reducing rooster mortality. In addition, a portion of roosters can be replaced, as practiced for broiler breeder flocks (called spiking). An important finding of this study, which may recommend the use of aviary systems for PS flock rearing to producers, is that such systems can contribute to elevated production of eggs, including hatching eggs, and higher fertilization, with unaltered chick quality.

## DISCLOSURES

The authors declare no conflicts of interest.

## Appendix Supplementary materials

Image, application 1

Image, application 2

Supplementary Data: No data were deposited in an official repository. All the data are available in the supplementary materials. Image, application 3

## References

[bib0001] Alexander, G. 2019. Multi-tier starts with aviary rear. Vencomatic recently opened the doors of its ‘first-of-a-kind’ Bolegg Starter rearing system in a well-attended open day. The Poultry Site. Accessed Jan. 2020. https://thepoultrysite.com/news/2019/10/multi-tier-starts-with-aviary-rear.

[bib0002] Ali A.B.A., Campbell D.L.M., Katcher D.M., Siegford J.M. (2016). Influence of genetic strain and access to litter on spatial distribution of 4 strains of laying hens in an aviary system. Poult. Sci..

[bib0003] Bakst M.R., Wishart G., Brullard J.P. (1994). Oviductal sperm selection, transport, and storage in poultry. Poult. Sci..

[bib0004] Berrang M.E., Frank J.F., Buhr R.J., Bailey J.S., Cox N.A., Mauldin J.M. (1997). Microbiology of sanitized broiler hatching eggs throught the egg production period. J. Appl. Poult. Res..

[bib0005] Brantsaeter M., Tahamtani F.M., Moe R.O., Hansen T.B., Orritt R., Nicol Ch., Janczak A.M. (2016). Rearing laying hens in aviaries reduces fearfulness following transfer to furnished cages. Front. Vet. Sci..

[bib0006] Campbell D.L.M., Makagon M.M., Swanson J.C., Siegford J.M. (2016). Laying hen movement in a commercial aviary: enclosure to floor and back again. Poult. Sci..

[bib0007] Chue J., Smith C.A. (2011). Sex determination and sexual differentiation in the avian model. FEBS J..

[bib0008] Clark C.E.F., Akter Y., Hungerford A., Thomson P., Islam M.R., Groves P.J., O'Shea C.J. (2019). The intake pattern and feed preference of layer hens selected for high or low feed conversion ratio. PLoS ONE.

[bib0009] Damaziak K., Musielak M., Musielak C., Riedel J., Gozdowski D. (2020). Reproductive performance and quality of offsprings of parental stock of layer hens after reading in open and closed aviary system. Poult. Sci..

[bib0010] European Communities (1999). Laying down minimum standards for the protection of laying hens. Off. J. Eur. Commun..

[bib0011] Freire R., Cowling A. (2013). The welfare of laying hens in conventional cages and alternative systems: first steps towards a quantative comparison. Anim. Welf..

[bib0012] Gunnarsson S., Keeling L.J., Svedberg J. (1999). Effect of reading factors on the prevelence of floor eggs, cloacal cannibalism and Feather pecking In commercial flocks of loose house laying hens. Br. Poult. Sci..

[bib0013] Haas E.N., Kemp B., Bolhuis J.E., Groothuis T., Rodenburg T.B. (2013). Fear, stress, and feather pecking in commercial white and brown laying hen parent-stock flocks and their relationship with production parameters. Poult. Sci..

[bib0014] Hendrix-Genetics (2014). Management Guide. Alternative Production System. http://www.cpif.org/wp-content/uploads/2014/04/ISA-Alternative-Productions-Management-Guide-copy.pdf.

[bib0015] Hendrix-Genetics (2020). ISA Brown Parent Stock Product Giude. http://colaves.com/pdf/isa_brown_PS_product_characteristics.pdf.

[bib0016] Hocking P.M., Bernard R. (2000). Effects of the age of male and female broiler breeders on sexual behaviour, fertility and hatchability of eggs. Br. Poult. Sci..

[bib0017] Janczak A.M., Torjesen P., Rettenbacher S. (2009). Environmental effects on steroid hormone concentrations in laying hens’ eggs. Acta Agr. Scand. A-Am..

[bib0018] Johnsen P.F., Vestergaard K.S., Norgaard-Nielsen G. (1998). Influence of early rearing conditions on the development of feather pecking and cannibalism in domestic fowl. Appl. Anim.l Behav. Sci..

[bib0019] Jones R.B., Larkins C., Hughes B.O. (1996). Approach/avoidance responses of domestic chicks to familiar and unfamiliar video images of biologically neutral stimuli. Appl. Anim. Behav. Sci..

[bib0020] Leonard M.L., Zanette L., Fairfull R.W. (1993). Early exposure to females affects interactions between male White Leghorn chickens. Appl. Anim. Behav. Sci..

[bib0021] Lodge J.R., Fechheimer N.S., Jaap R.G. (1971). The relationship of *in ovo* sperm storage interval to fertility and embryonic survival in the chicken. Biol. Reprod..

[bib0022] Luiting P., Schrama J.W., Van der Hel W., Urff E.M. (1991). Metabolic differences between white leghorns selected for high and low residual feed consumption. Br. Poult. Sci..

[bib0023] Matthews W.A., Summer D.A. (2015). Effects of housing system on the costs of commercial egg production. Poult. Sci..

[bib0024] Merle, R., S. Koesters, C. Suerie, A. Ovelhey, and L. Kreienbrock. 2009. Mortality in laying hens – a comparison of different housing systems. Proceedings of the XIV ISAH Congress 2009 1, XIV ISAH Congress 2009 of the International Society for Animal Hygiene “Sustainable Animal Husbandry: Prevention is better than cure” At: Vechta, Germany.

[bib0025] Moe R.O., Guemene D., Bakken M., Larsen H.J.S., Shini S., Lervik S., Skjerve E., Michel V., Tauson R. (2010). Effects of housing conditions during the rearing and laying period on adrenal reactivity, immune response and heterophil to lymphocyte (H/L) ratios in laying hens. Anim..

[bib0026] Riber A.B., Casey-Trott C., Herskin M.S. (2018). The influence of keel bone damage on welfare of laying hens. Front. Vet. Sci..

[bib0027] Sørensen P., Okeno T., Buitenhuis A.J. (2017). The motivation of hens to lay eggs on the floor in non-cage systems has a heritable background. Eur. Poult. Sci..

[bib0028] Tahamtani F.M., Hansen T.B., Orritt R., Nicol C., Moe R.O., Janczak A.M. (2014). Does rearing laying hens in aviaries adversely affect long-term welfare following transfer to furnished cages?. PLoS ONE.

[bib0029] Tona K., Bamelis F., De Ketelaera B., Bruggeman V., Moraes V.M.B., Buyse J., Onagbesan O., Decuypere E. (2003). Effects of egg storage time on spread of hatch, chick quality, and chick juvenile growth. Poult. Sci..

[bib0030] TIBCO Software Inc (2017). Statistica (Data Analysis Software System), Version 13. http://statistica.io.

[bib0031] Van den Brand H., Sosef M.P., Lourens A., van Harn J. (2016). Effects of floor eggs on hatchability and later life performance in broiler chickens. Poult. Sci..

[bib0032] Van de Weerd H.A., Elson A. (2006). Rearing factors that influence the propen sity for injurious feather packing in laying hens. Worlds Poult. Sci. J..

[bib0033] Weeks C.A., Lambton S.L., Williams A.G. (2016). Implications for welfare, productivity and sustainability of the variation in reported levels of mortality for laying hen flocks kept in different housing systems: a meta-analysis of ten studies. PLoS ONE.

[bib0034] Xin H., Gates R.S., Puma M.C., Ahn D.U. (2002). Drinking water temperature effects on laying hens subjected to warm cyclic environments. Poult. Sci..

[bib0035] Yang N., Wu C., McMillan I. (1989). New mathematical model of poultry egg production. Poult. Sci..

