# Supplementary material for: Effect of different rearing system on eggs production, hatchability, and offspring quality in layer breeders
Source: Poult Sci. 2021 Mar 11;100(6):101101. doi: 10.1016/j.psj.2021.101101 (PMC8131713; doi:10.1016/j.psj.2021.101101)
Supplement: Supplementary file 1 [file mmc1.pdf]

Poultry Science

Effects of different rearing system on eggs production, hatchability, and offspring quality in layer breeders

K. Damaziak, M. Musielak, C. Musielak, J. Riedel, D. Gozdowski, W. Grzybek

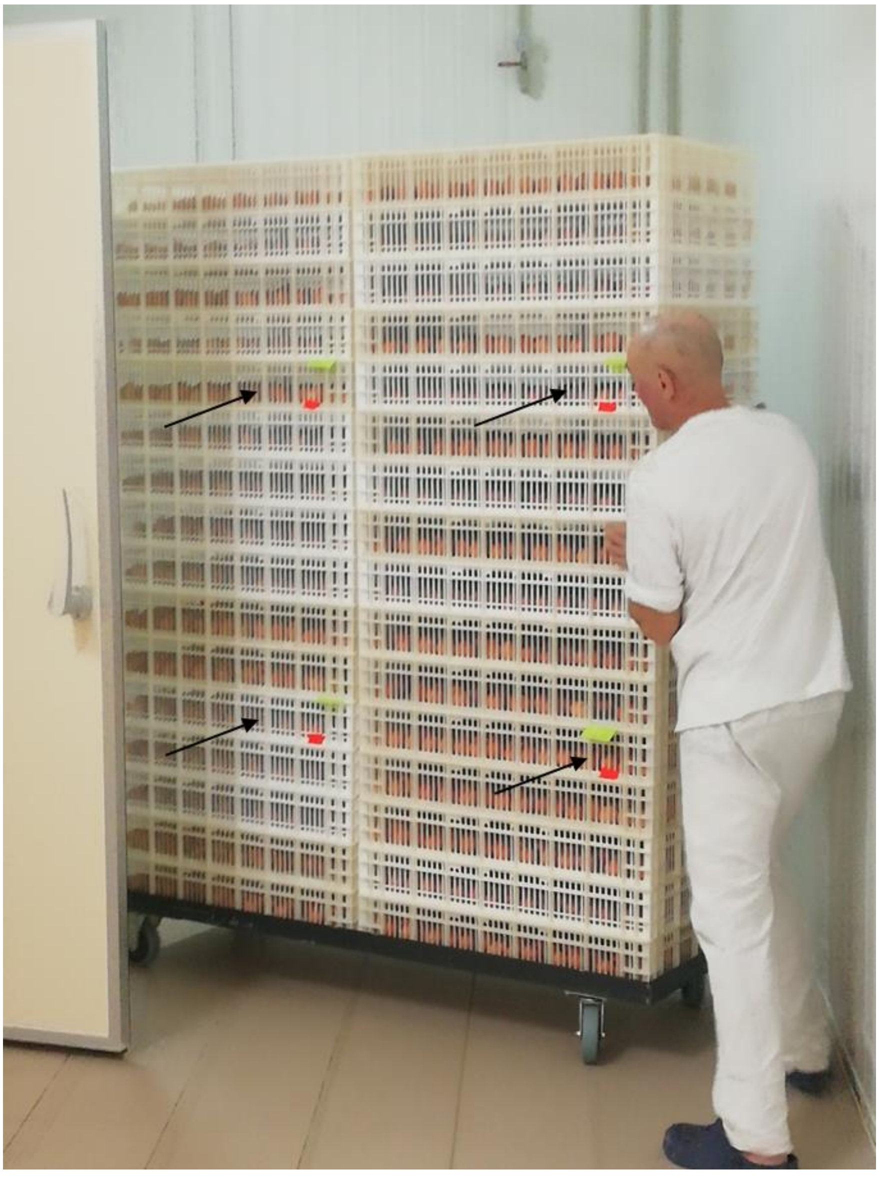

Supplementary Figure S1. Stroller with marked (arrow) hatching baskets selected to hatch analysis
