# Supplementary material for: Effect of different rearing system on eggs production, hatchability, and offspring quality in layer breeders
Source: Poult Sci. 2021 Mar 11;100(6):101101. doi: 10.1016/j.psj.2021.101101 (PMC8131713; doi:10.1016/j.psj.2021.101101)
Supplement: Supplementary file 2 [file mmc2.docx]

**Effects of different rearing system on eggs production, hatchability, and offspring quality in layer breeders**

K. Damaziak, M. Musielak, C. Musielak, J. Riedel, D. Gozdowski, Weronika Grzybek

**Supplementary Table S1**

Parameters of Yang's model for the analysis of the parental stocks laying rate after rearing in battery cages and aviary system

| Model: laying rate = (a*Exp(day*b))/(1+Exp(c*(day-d))) | | | | | | | |
| --- | --- | --- | --- | --- | --- | --- | --- |
| Rearing system | Parameter | Score | Error | The value of time | *P* - value | Lower confidence | Upper confidence |
| Aviary | a | 0.99518 | 0.004032 | 246.8113 | 0.00 | 0.98724 | 1.00311 |
|  | b | -0.00044 | 0.000020 | -21.3664 | 0.00 | -0.00048 | -0.00040 |
|  | c | -0.28512 | 0.009520 | -29.9482 | 0.00 | -0.30384 | -0.26639 |
|  | d | 23.99272 | 0.134560 | 178.3044 | 0.00 | 23.72802 | 24.25743 |
| Cages | a | 0.99749 | 0.002542 | 392.3665 | 0.00 | 0.99249 | 1.00250 |
|  | b | -0.00080 | 0.000013 | -60.2305 | 0.00 | -0.00082 | -0.00077 |
|  | c | -0.26707 | 0.005331 | -50.0974 | 0.00 | -0.27756 | -0.25658 |
|  | d | 21.25457 | 0.086026 | 247.0720 | 0.00 | 21.08534 | 21.42380 |

a = asymptotic value of egg production at the peak of egg-laying; b = rate of production decrease after the peak (eggs/hen-day decrease per week); c = reciprocal indicator of the variation in wk of production of first egg; d = mean wk of egg production at sexual maturity
